# Pharmacological Prevention of Peri-, and Post-Procedural Myocardial Injury in Percutaneous Coronary Intervention

**DOI:** 10.2174/157340308785160598

**Published:** 2008-08

**Authors:** Hideki Ishii, Tetsuya Amano, Tatsuaki Matsubara, Toyoaki Murohara

**Affiliations:** a Department of Cardiology, Nagoya University Graduate School of Medicine; b Department of Internal Medicine, School of Dentistry, Aichi-Gakuin University, Nagoya, Japan

**Keywords:** Coronary artery disease, percutaneous coronary intervention, myocardial injury, pharmacology.

## Abstract

In recent years, percutaneous coronary intervention (PCI) has become a well-established technique for the treatment of coronary artery disease. PCI improves symptoms in patients with coronary artery disease and it has been increasing safety of procedures. However, peri- and post-procedural myocardial injury, including angiographical slow coronary flow, microvascular embolization, and elevated levels of cardiac enzyme, such as creatine kinase and troponin-T and -I, has also been reported even in elective cases. Furthermore, myocardial reperfusion injury at the beginning of myocardial reperfusion, which causes tissue damage and cardiac dysfunction, may occur in cases of acute coronary syndrome. Because patients with myocardial injury is related to larger myocardial infarction and have a worse long-term prognosis than those without myocardial injury, it is important to prevent myocardial injury during and/or after PCI in patients with coronary artery disease. To date, many studies have demonstrated that adjunctive pharmacological treatment suppresses myocardial injury and increases coronary blood flow during PCI procedures. In this review, we highlight the usefulness of pharmacological treatment in combination with PCI in attenuating myocardial injury in patients with coronary artery disease.

## INTRODUCTION

Percutaneous coronary intervention (PCI) relieves cardiac ischemia, resulting in improvement of symptoms in patients with coronary artery disease (CAD). Although PCI has been widely performed in the management of CAD, non-Q wave myocardial infarction (MI) can occur in 10-40% of patients after elective PCI [1-5]. This phenomenon can be detected by changes of electrocardiography (ECG) and elevation of cardiac enzymes such as troponin-I and T, creatine kinase (CK), creatine kinase-myocardial band isoenzyme fraction (CK-MB), and so on. There are many reports that myocardial damage during PCI is associated with increase in risk of major in-hospital complications and major adverse clinical events, even if it is small [5-11]. In many cases, cardiac enzyme elevation results from various procedural events including side branch closure and distal thromboembolism, which can be confirmed by coronary angiography [12]. However, in some cases, cardiac enzyme elevations occur even without discernible complications. Dilatation of plaque and vessel wall, which include lipid, matrix, endothelial cells and platelet thrombus, may induce microcirculatory embolism after angioplasty [13,14]. Therefore, coronary dilation and reduction of oxidative stress, inflammation, and platelet activation triggered by medications may thus attenuate minor cardiac necrosis during and after PCI, and reduce cardiac enzyme marker release. Recently, coronary stent implantation, which features a lower rate of restenosis during follow-up period than balloon angioplasty alone, has becomes a popular procedure [15,16]. However, it is associated with a higher incidence of cardiac enzyme elevation than balloon angioplasty, and use of intracoronary metallic stent increases platelet activation [17,18]. Therefore, preventing cardiac enzymate elevations is now becoming of much greater importance.

In patients with acute coronary syndrome, including unstable angina and acute myocardial infarction (AMI), myocardial reperfusion injury at the beginning of myocardial reperfusion may occur, resulting in myocardial tissue damage and cardiac dysfunction. In such situations, ECG, myocardial contrast echocardiographical and angiographical findings after successful PCI can detect myocardial reperfusion at the microcirculatory level and myocardial damage [19-22].

Combined use of pharmacological intervention in addition to PCI may moderate microcirculatory impairment and eventually reduce the incidence and/or severity of infarction, resulting in better clinical outcomes after PCI. In this review, the usefulness of pharmacological treatment in combination with PCI in attenuating myocardial injury in patients with CAD is highlighted.

## GLYCOPROTEIN IIb/IIIa INHIBITORS

The platelet surface glycoprotein IIb/IIIa receptors play an important role in the final common pathway leading to platelet aggregation [23,24]. Platelet deposition and aggregation are activated during PCI, particularly coronary stent implantation [13,14,25]. This phenomenon may induce platelet aggregation, which is related to acute coronary closure after procedures. Glycoprotein IIb/IIIa inhibitors disperse platelet deposition and also protect coronary microcirculation from embolization of platelet thrombi, which is sometimes induced by coronary balloon inflation and stent implantation. Therefore glycoprotein IIb/IIIa inhibitors have beneficial effects on preventing myocardial injury in these contexts. 

In many large trials, some glycoprotein IIb/IIIa inhibitors have been reported to be effective in reducing ischemic complications in patients treated with PCI [24-30]. Of various glycoprotein IIb/IIIa inhibitors, abciximab has been most often used in trials for evaluation of clinical outcomes in patients undergoing PCI. In the EPIC trial, abciximab reduced the incidence of acute ischemic events by 35% in patients undergoing PCI [26]. However, it increased the incidence of major bleeding complications. In that study, concurrent administration of high doses of heparin was used. In the EPILOG trial [27], abciximab also had beneficial effects in reducing the risk of acute ischemic complications after PCI procedures. In bleeding complications, there were no significant differences among the group assigned to placebo with standard-dose heparin, the group assigned abciximab with low-dose, weight-adjusted heparin, and the group assigned abciximab with standard-dose, weight-adjusted heparin, though minor bleeding events were more frequently seen in the group assigned abciximab with standard-dose heparin. In a sub-analysis of the EPISTENT trial [29], abciximab significantly reduced angiographic complications including major and minor dissections, distal embolization, thrombus postprocedure, side branch or other vessel occlusion, residual stenosis >50%, transient coronary occlusion, and Thrombolysis In Myocardial Infarction (TIMI) final flow <3 during coronary stenting, compared with placebo [30]. In that study, abciximab also significantly reduced the incidence of CK-MB elevation in patients without angiographic complications.

According to AMI patients undergoing PCI, it has been reported that administration of glycoprotein IIb/IIIa inhibitors improves myocardial reperfusion [31,32]. In the ADMIRAL trial [31], early administration of abciximab (a 0.25 mg/kg abciximab bolus before catheterization followed by a 12-hour infusion of 0.125 μg/kg/min) significantly improved coronary patency before and after stenting in patients with AMI, compared to placebo administration. A meta-analysis of six randomized trials showed that early administration of glycoprotein IIb/IIIa inhibitors in patients with ST-elevation-AMI improved coronary patency after PCI with favorable trends for clinical outcomes [32].

### Bivalirudin

Bivalirudin is a direct thrombin inhibitor. Some studies found that bivalirudin use to be noninferior to treatment with heparin plus glycoprotein IIb/IIIa inhibitors [33,34]. Furthermore, bleeding complications were less frequently seen in patients with periprocedural bivalirudin therapy than in patients with heparin plus glycoprotein IIb/IIIa inhibitors [34]. Thus, in this context, bivalirudin has a beneficial effect.

### Thienopyridine

Thienopyridine compounds such as ticlopidine and clopidogrel inhibit the platelet adenosine diphosphate receptor, resulting in blocking platelet aggregation. Dual antiplatelet therapy with aspirin and thienopyridine compound is widely used to prevent stent thrombosis and ischemic complication after PCI procedure [35, 36]. However, there are few studies focusing on the effect of pre-treatment with thienopyridine on preventing procedural related myocardial injury in patients undergoing PCI. 

One retrospective report suggests that beginning ticlopidine therapy several days prior to elective stent implantation was associated with a reduced risk of procedural non-Q-wave myocardial infarction [37]. On the other hand, a prospective study reported that pretreatment with clopidogrel failed to decrease post-procedural elevation of troponin I and CK-MB [38]. It is also controversial whether there is an additive anti-platelet effect of ticlopidine in combination with glycoprotein IIb/IIIa inhibitors.

### Adenosine

It is well known that brief episodes of ischemia in patients with repetitive balloon inflation during elective PCI relieve angina attack and ST elevation in ECG and that effects of transient episodes of angina protect myocardium from ischemic damage in patients with acute coronary syndrome [39-45]. These cardioprotective effects are called preconditioning phenomenon. Adenosine plays a key role in preconditioning of myocardium [46,47], and thus might therefore have a beneficial effect in preventing myocardial necrosis following PCI, in addition to its vasodilator effect. 

A small clinical trial consisting of 28 patients (16 in the control group and 12 in the adenosine group) showed that the intracoronary administration of adenosine (1 mg/min in the right coronary artery or 2 mg/min in the left coronary artery) over a 10-minute period during PCI reduced release of serum CK-MB 24 hours after PCI [48]. 

Hanna *et al.* reported that intracoronary administration of adenosine prevented no reflow phenomenon during rotational atherectomy [49]. This report is very important because that slow flow and no reflow phenomenon is a common complication of rotational atherectomy [50,51].

It has been reported that in AMI patients treated with PCI, intracoronary administration of 4 mg adenosine in 2 ml saline into the distal bed of a totally occluded vessel prevented no-reflow phenomenon, and improved LV function a week after PCI [52].

### Nitric Oxide

Nitric oxide has been used for the relief of attacks of angina. Nitrates improve the myocardial oxygen supply-to-demand ratio in ischemic zones and relax coronary smooth muscle cells, resulting in suppression of coronary vasospasms [53-55].

Kurz *et al.* examined whether the intravenous administration of nitroglycerin for 12 hours after elective coronary stenting would decrease the incidence of angina and minor myocardial necrosis, as detected by cardiac troponin I increase, in a prospective, single center study including 100 patients [56]. Although the incidence of post-procedural chest pain did not differ between the two groups, the rate of occurrence of minor myocardial necrosis was significantly reduced in the nitroglycerin group.

Amit *et al.* reported that the intracoronary administration of nitroprusside (60 μg), selectively injected into distal to the occlusion of the infarct-related artery, did not prevent the no-reflow phenomenon in patients with AMI, treated with primary PCI, although nitroprusside improved clinical outcomes at 6 months after PCI [57]. 

### Nicorandil

Pharmacological treatment with ATP-sensitive potassium channel openers have similar cardioprotective effects like ischemic preconditioning [45,58]. As mentioned above, ischemic preconditioning effects can help avoid complications during PCI and have important therapeutic implications.

Nicorandil, a hybrid of adenosine triphosphate-sensitive K channel opener and nitrates, has vasodilatory effects and thus increases coronary blood flow particularly in small vessels, prevents vasospasms and improves microvascular circulation [58-61], and cardiac sympathetic nerve activity, and thereby prevents slow flow in patients with ischemic heart disease [62]. For these reasons, nicorandil is now used as a pharmacological adjunct to PCI. Murakami *et al.* investigated whether intravenous 6-hour nicorandil infusion (a dose of 2 μg/kg/min starting just before PCI) would reduce the incidence of minor cardiac marker elevation in 192 patients undergoing elective coronary stenting [63]. Nicorandil treatment reduced the incidence of elevation of cardiac markers and decreased the levels of such markers in serum after PCI with stent implantation. Kuwabara *et al.* found that in 48 patients treated with elective PCI, intracoronary and intravenous nicorandil during PCI improved the recovery of myocardial fatty acid utilization on evaluation by iodine-123-beta-methyl-p-iodophenyl-pentadecanoic acid single photon emission computed tomography (I-123 BMIPP SPECT), and that this improvement was related to better left ventricular systolic function [64]. Iwasaki *et al.* showed that preventive effect on the incidence of slow or no reflow during rotational atherectomy was much more seen in patients treated with intracoronary nicorandil, compared with verapamil [65]. 

Ito *et al.* performed a prospective, single-center study including 81 patients with a first anterior AMI who received successful PCI and showed effectiveness of intravenous nicorandil (6 mg for 24 hours after bolus injection at a dose of 4 mg) on preventing no-reflow phenomenon microvascular function as assessed by intracoronary myocardial contrast echocardiography [66]. We reported that the addition of 12 mg of nicorandil intravenously just before direct PCI accelerated ST-segment resolution and improved epicardial flow in patients with ST-elevation AMI [67].

Nicorandil dose-dependently increases coronary artery blood flow and improves other physiological parameters [68], although administration of it, particularly in intracoronary fashion, may induce ventricular arrhythmia [69]. Further study is thus needed to determine optimal methods of administration and doses of this agent.

Diazoxide is also known as an ATP-sensitive potassium channel opener. Although experimental studies have shown cardioprotective effects of diazoxide, there are limited clinical data in PCI cases. 

### Beta-Blockers

Beta-blocker therapy in patients with ischemic heart disease has been widely reported. Some studies examined whether beta-blocker reduced clinical complications in patients treated with PCI. Some experimental studies have shown that intravenous administration of beta-blockers prior to coronary artery occlusion favorably reduced electrocardiographic and enzymatic parameters [70-72].

Sharma *et al.* performed a prospective observational but non-randomized study including 1675 consecutive patients undergoing PCI, and showed that prior administration of beta-blockers before PCI had a cardioprotective effect in limiting CK-MB release after PCI [73]. On the other hand, Ellis *et al.* reported that there appeared to be no relationship between prior beta-blockers use and rise in CK-MB in a large and consecutive cohort of 6200 patients undergoing PCI [74]. There were important differences between these two studies in the statistical methods used to adjust differences between patients treated with or without beta-blockers. However, neither was a randomized study with assignment of beta-blockers or placebo to patients. 

A recent double-blind clinical trial showed that the intracoronary administration of propranolol (15 μg/kg), selectively delivered before first balloon inflation *via* an intracoronary catheter with the tip distal to the coronary lesion, significantly reduced the incidence of CK-MB elevation and troponin-T elevation after PCI and improve short-term clinical outcomes, compared to placebo [75]. Further study is needed to assess the efficacy of beta-blockers in the prevention of myocardial injury. 

## ANGIOTENSIN CONVERTING ENZYME INHIBITORS

Treatment with angiotensin converting enzyme (ACE) inhibitors induces accumulation of bradykinin, reduces plasma angiotensin II levels and decreases plasma plasminogen activator inhibitor-1 (PAI-1), a risk factor for myocardial infarction [76,77]. These mechanisms are expected to be potentially beneficial in patients undergoing PCI. Particularly increasing bradykinin has been experimentally found to trigger preconditioning effects experimentally [78].

Recently, it has been reported that enalaprilat increased coronary blood flow, and that pretreatment with enalaprilat resulted in less ST-segment shift and less chest pain during the first balloon inflation in a prospective randomized study including 22 patients undergoing elective PCI [79]. 

### 3-hydroxy-3-methylglutaryl co-enzyme A (HMG-CoA) Reductase Inhibitors: Statins

3-hydroxy-3-methylglutaryl co-enzyme A (HMG-CoA) reductase inhibitors (statins) are widely used for treatment with hypercholesterolemia and/or coronary artery disease [80,81]. It is well known that statins have non-lipid-lowering pleiotropic effects including anti-thrombotic effects, improvement of vascular endothelial function, and reduction of oxidative stress and inflammation [82,83].

In nonrandomized observational studies, preoperative treatment with statins before elective PCI was associated with lower levels of periprocedural CK and CK-MB elevation [84,85]. Furthermore, randomized trials have indicated that statin administration before elective PCI reduces the rate of myocardial injury after PCI. In the Atorvastatin for Reduction of Myocardial Damage during Angioplasty (ARMYDA) study, 153 patients scheduled for elective PCI were randomized to receive atorvastatin (40 mg/day) or placebo 7 days before the procedure [86]. Atorvastatin treatment significantly reduced post-procedural release of several markers of myocardial damage, including myoglobin, troponin I, and CK-MB, compared with the control. Briguori *et al.* investigated whether pre-procedural statin administration started at least 3 days before elective PCI was effective in preventing elevation of myocardial injury after the procedure and found that the incidence of CK-MB elevation >5 times the upper limit of normal was approximately 50% lower in patients treated with pre-procedural statin administration [87].

In AMI cases, we recently reported that ST segment resolution >50% on electrocardiography after direct PCI was much more observed in patients receiving chronic statin treatment before the onset of AMI than in patients without statin and that patients treated with statins had a lower peak CK levels [88]. Iwakura *et al.* demonstrated the effectiveness of chronic statin pretreatment before AMI in decreasing the incidence of the no-reflow phenomenon as assessed by intracoronary myocardial contrast echocardiography [89].

Many trials have demonstrated decreases in the incidence of myocardial injury after PCI for coronary artery disease in patients treated with pre-procedural statins. However, because these trials consisted of small study samples, larger multi-center studies are warranted to corroborate their findings.

## CONCLUSIONS

From various mechanisms, pharmacological intervention increases coronary blood flow, suppresses myocardial damage, and prevents complications during and after PCI procedures.

PCI may induce thromboembolism and/or atheroembolism in the distal coronary bed. The effect of treatment with glycoprotein IIb/IIIa inhibitors, bivalirudin, and thienopyridine compounds is thought to block platelet aggregation and thus better prevention of procedural thrombus formation. Pre-treatment with statins may prevent microvascular plugging due to atheroembolism. PCI may also induce microvascular spasms and/or edema. For these mechanisms, adenosine, nitric oxide, and nicorandil play key roles. Cardioprotective effects mimicking preconditioning effects may decrease myocardial injury. From this point, adenosine, ATP-sensitive potassium channel openers, and ACE inhibitors may be useful. It is also important that oxygen free-radical production is prevented and endothelial functions are preserved by some medications. The ability of pharmacological treatment to prevent peri-, and post-procedural myocardial injury when added to PCI, thus has important therapeutic implications.

## Figures and Tables

**Table 1:** Adjunctive Pharmacological Therapy in Combination with Percutaneous Coronary Intervention

Cardioprotective Strategy and Trial	No. of Patients	Detail of Study	Findings
**Glycoprotein IIb/IIIa inhibitors**			
EPIC [26]	2099	A bolus and an infusion of placebo, a bolus of abciximab and an infusion of placebo, or a bolus and an infusion of abciximab in patients undergoing high-risk percutaneous coronary revascularization procedures	As compared with placebo, the abciximab bolus and infusion resulted in a 35 percent reduction in the rate of the adverse cardiac events (12.8% vs. 8.3%, p = 0.008), whereas a 10 percent reduction was observed with the abciximab bolus alone (12.8% vs. 11.5%, p = 0.43).
EPILOG [27]	2792	Standard-dose, weight-adjusted heparin (initial bolus of 100 U per kilogram of body weight); abciximab with low-dose, weight-adjusted heparin (initial bolus of 70 U per kilogram); or placebo with standard-dose, weight-adjusted heparin in patients undergoing urgent or elective PCI	Abciximab treatment, together with weight-adjusted heparin, markedly reduces the risk of acute ischemic complications in patients undergoing PCI. Although there were no significant differences among the groups in the risk of major bleeding, minor bleeding was more frequent among patients receiving abciximab with standard-dose heparin.
EPISTENT [30]	1587	Treatment with abciximab (0·25 mg/kg before intervention, followed by an infusion of 0·125 µg/kg/min for 12 h) or placebo in patients undergoing stent implantation.	Abciximab (compared with placebo) significantly reduced the incidence of CK-MB elevation >3 times normal in those without any angiographic complications (6.5% vs. 10.7%; p = 0.007).
ADMIRAL [31]	300	Abciximab (a bolus of 0·25 mg/kg before intervention, followed by a 12-hour infusion of 0·125 µg/kg/min) or placebo in patients with AMI	Treatment with abciximab significantly improved coronary patency before and after stenting in patients with AMI, compared to placebo administration (16.8% vs. 5.4%, p = 0.01 and 95.1% vs. 86.7%, p = 0.04, respectively).
**Bivalirudin**			
Feldman, DN *et al.* [34]	2504	Bivalirudin (a 0.75-mg/kg intravenous bolus followed by an infusion of 1.75 mg/kg per hour for the duration of the PCI procedure) or abciximab (a 0.25-mg/kg bolus and a 0.125 µg/kg /min infusion for 12 hours) in patients undergoing PCI with drug-eluting stents	The incidence of cardiac adverse events was similar in the bivalirudin and heparin plus GP IIb/IIIa inhibitor groups. There was a lower incidence of major (0.7% vs 1.9%, *P* = .012) and minor bleeding (9.6% vs 15.6%, *P* < .001) in the bivalirudin versus heparin plus GP IIb/IIIa inhibitor group.
**Thienopyridine**			
Steinhubl, SR *et al.* [37]	175	Ticlopidine pretreatment: ≥3 days before the procedure, 1 to 2 days, or <1 day before stent implantation	Ticlopidine pretreatment of ≥ 3 days was associated with a significant reduction in the risk of non–Q-wave MI (unadjusted odds ratio 0.18, 95% confidence interval = 0.04 to 0.78, p = 0.01) compared with pretreatment of <3 days.
**Adenosine**			
ADELINE pilot trial [48]	28	Intracoronary 1 mg/min in the right coronary artery or 2 mg/min in the left coronary artery over a 10 minute period or placebo in patients undergoing elective PCI	There was a significant difference in CK-MB increase from baseline to 24 hours after the PCI between the adenosine treated and the control group (1.15 vs. 7.36 µg/l, p = 0.047).
Marzilli, M *et al.* [52]	54	Intracoronary adenosine (4 mg) or saline in AMI patients undergoing primary PCI	The no-reflow phenomenon was seen in 1 adenosine patient and in 7 saline patients (p = 0.02). A Q-waveMI developed in 59.3% of the adenosine group and in 85.2% of the saline group (p = 0.04).
**Nitric oxide**			
Kurz, DJ *et al.* [56]	100	Intravenous nitroglycerin or placebo during 12 hours after stenting in patients with stable or unstable angina	The occurrence of minor myocardial necrosis was 5% in the nitroglycerin group and 22% in the placebo group (p = 0.036).
Amit, G *et al.* [57]	98	Nitroprusside (60 µg) selectively injected into the infarct-related artery, distal to the occlusion or placebo in ST-elevation AMI patients	Selective intracoronary administration of nitroprusside failed to improve myocardial perfusion, assessed by angiography or ECG. However, nitroprusside improved clinical outcomes at 6 months after PCI.
**Nicorandil**			
Murakami, M *et al.* [63]	192	Intravenous nicorandil (2 µg/kg/min administered just before PCI and continued for 6 hours) or control in patients undergoing elective coronary stenting	The incidence of CK-MB elevation after PCI was significantly lower in patients treated with nicorandil than in those without nicorandil (8.8% vs. 21.8%, p <0.05). The mean peak CK-MB level postprocedure was 13.4 IU in patients with nicorandil and 16.5 IU in those without nicorandil (p <0.01).
Kuwabara, Y *et al. * [64]	48	Intracoronary and intravenous nicorandil during PCI or control in patients undergoing elective PCI	Nicorandil improved the recovery of myocardial fatty acid utilization and cardiac function after PCI, quantitatively evaluated by means of iodine-123-beta-methyl-p-iodophenyl-pentadecanoic acid single photon emission computed tomography.
Ito, H *et al.* [66]	81	Intravenous nicorandil (6 mg for 24 hours after bolus injection at a dose of 4 mg) or control in patients with anterior AMI	The frequency of no reflow phenomenon detected by myocardial contrast echocardiography was significantly lower in the nicorandil group than in the control group (15% vs. 33%, p < 0.05).
Ishii, H *et al. * [67]	368	Intravenous 12 mg dose of nicorandil or placebo in patients with ST elevation AMI	Postprocedural TIMI 3 flow was obtained in 89.7% of the nicorandil group and in 81.4% of the placebo (hazard ratio 1.99, 95% CI 1.09-3.65; p = 0.025). Corrected TIMI frame count was 21.0 ± 9.1 in the nicorandil group and 25.1 ± 14.1 in the placebo group (p = 0.0009). ST-segment resolution >50% was observed in 79.5% of the nicorandil group and in 61.2% of the placebo group (hazard ratio 2.45, 95% CI 1.54-3.90; p = 0.0002).
**Beta-blocker**			
Sharma, SK *et al.* [73]	1675	Beta-blocker administration before PCI or no beta-blocker administration	The incidence of CK-MB elevation after PCI was significantly lower in patients on beta-blocker than in those without beta-blockers (13.2% vs. 22.1%, p <0.001). The mean peak CK-MB level postprocedure was 29 IU in patients on beta-blocker and 38 IU in those without beta-blockers (p <0.002).
Ellis, SG *et al.* [74]	6200	Beta-blocker administration before PCI or no beta-blocker administration	There was no difference in either maximum CK rise or maximum CK-MB rise after PCI between patients with and without beta-blocker after adjustment for significant differences in baseline characteristics.
Wang, FW *et al.* [75]	150	Propranolol (15 µg/kg) injected into the coronary artery through the dilatation catheter with the tip distal to the coronary lesion or placebo	The incidence of CK-MB elevation after PCI was seen in 17% of patients treated with propranolol and 36% of patients with placebo (p = 0.01). Patients in the propranolol group had lower incidence of troponin T elevation than patients in the placebo group (13% vs. 33%, p = 0.005).
**Angiotensin converting enzyme inhibitor**			
Leesar, MA *et al.* [79]	22	Intracoronary 0.75 mg dose of enalaprilat or placebo in patients with chronic stable angina	In the placebo group, the ST-segment shift was greater during the first balloon inflation than during the second and third inflations, both on the intracoronary ECG (21.0 ± 2.8 mm vs. 13.0 ± 2.0 mm and 13.0 ± 2.0 mm, p < 0.05) and on the surface ECG (16.0 ± 4.0 mm vs. 10.0 ± 2.0 mm and 9.0 ± 2.0 mm, p < 0.05).
**Statin**			
ARMYDA [86]	153	Atorvastatin (40 mg/day) or placebo 7 days before elective PCI for chronic stable angina	The incidence of myocardial infarction by CK-MB determination was significantly lower in the atrvastatin group than in the placebo group (5% vs. 18%, p = 0.025).
Briguori, C *et al.* [87]	451	Statin administration at least 3 days before elective PCI or no statin administration	Median CK-MB peak after PCI was significantly lower in the statin group than in the control group (1.70 vs. 2.20 ng/ml, p = 0.015). The incidence of a large non-Q-wave myocardial infarction was also lower in the statin group (8.0% vs. 15.6%: odds ratio 0.47; 95% confidence interval = 0.26-0.86, p = 0.012).
Ishii, H *et al. * [88]	386	Chronic statin therapy for ≥1 month before the onset of a first AMI or control	Patients in the statin group had a significantly lower maximum CK level (2300 vs. 3538 IU/ml, p = 0.015) and a lower corrected TIMI frame count (18.8 vs. 24.2, p = 0.017) than patients in the non-statin group.
Iwakura, K *et al.* [89]	293	Chronic statin treatment before admission for AMI or control	Patients in the statin group had lower incidence of the no-reflow detected by intracoronary myocardial contrast echocardiography than patients in the control (9.1% vs. 34.6%, p = 0.003).
